# Some Water Uses Well to Remember

**Published:** 1897-08

**Authors:** 


### Some Water Uses Well to Remember.

The *Phrenological Journal* gives the following useful hints on the applications of water in severe attacks of illness. The adult members of a family should keep them in mind for an emergency:

A strip of flannel or a soft napkin, folded lengthwise and dipped in hot water and wrung out, and then applied around the neck of a child that has the croup, will usually bring relief in a few minutes.

A proper towel folded several times, and dipped in hot water, quickly wrung and applied over the site of toothache or neuralgia, will generally afford prompt relief.

This treatment for colic has been found to work like magic.

Nothing so promptly cuts short a congestion of the lungs, sore throat or rheumatism, as hot water, when applied early in the case and thoroughly.

Hot water taken freely half an hour before bedtime is an excellent cathartic in the case of constipation, while it has a soothing effect upon the stomach and bowels.

This treatment, continued a few months, with the addition of a cup of hot water slowly sipped half an hour before each meal, with proper attention to diet, will cure most cases of dyspepsia.

Ordinary headaches almost always yield to the simultaneous application of hot water to the feet and back of the neck.—*Scientific American.*

